# Enhancing HIV commodity reporting using real-time antiretrovirals stock status monitoring tool in an HIV-accredited regional referral hospital, Kampala, March 2024 – August 2024

**DOI:** 10.4102/jphia.v17i1.1666

**Published:** 2026-08-05

**Authors:** Tracy Maureen Rutogire, Samuel Gidudu, Gloria Bahizi, Jackson Were, Irene B. Kyamwine, Lilian Bulage, Alex Riolexus Ario

**Affiliations:** 1 Uganda Public Health Fellowship Program, National Institute of Public Health, Kampala, Uganda; 2 Department of Public Health and Environment, Kampala Capital City Authority, Kampala, Uganda; 3 Department of Global Health Security, Baylor Foundation Uganda, Kampala, Uganda; 4 Department of Laboratory Diagnostics, Mulago National Referral Hospital, Kampala, Uganda

**Keywords:** HIV commodity reporting, real-time ARV stock status monitoring, RASS, continuous quality improvement, CQI, staff orientation, WhatsApp reminders

## Abstract

**Background:**

Timely reporting of human immunodeficiency virus (HIV) commodity stock status is critical for effective supply chain management. However, only four (12%) of 31 HIV-accredited sites in the region submitted all weekly reports in quarter one 2024 using the Real-time Antiretrovirals (ARV) Stock Status Monitoring (RASS) tool.

**Aim:**

To improve weekly reporting from 0% to 100% by August 2024 at a non-reporting HIV-accredited regional referral hospital using a continuous quality improvement (CQI) approach.

**Setting:**

The project was conducted at a regional referral hospital in Kampala, Uganda, an HIV-accredited facility serving approximately 1354 HIV patients as of March 2024.

**Methods:**

A CQI team conducted a baseline assessment in March 2024 using RASS dashboard data and identified non-reporting causes using fishbone and 5 Whys techniques. Interventions, implemented from April 2024 to July 2024, included orienting new staff and introducing WhatsApp reminders. Progress was monitored weekly using the *Plan–Do–Check–Act (PDCA)* cycle, with data analysed monthly to assess trends.

**Results:**

Baseline reporting was 0% in March 2024 because of a lack of staff orientation. Orientation of two RASS users in April 2024 increased reporting to 80% by May 2024, and with WhatsApp reminders in June 2024, it reached 100% and was sustained through August 2024.

**Conclusion:**

Staff orientation and WhatsApp reminders were associated with improved HIV commodity reporting at the study site. While these interventions appear promising, further evaluation across multiple sites and under controlled conditions is needed to confirm their effectiveness. Institutionalising such low-cost strategies may offer potential benefits for strengthening HIV commodity stock management.

**Contribution:**

These low-cost, scalable interventions can enhance HIV commodity management across HIV-accredited sites, thus supporting 95-95-95 HIV targets.

## Introduction

The human immunodeficiency virus (HIV) epidemic remains a public health priority in the region, with the country working towards the Joint United Nations Programme on HIV/AIDS (UNAIDS) 95-95-95 goals by 2030.^1^ Effective supply chain management of antiretrovirals (ARV) and HIV test kits is essential to prevent stock-outs, which have repeatedly disrupted HIV care in the country.^2,3^ To strengthen supply chain management, the Monitoring and Evaluation Technical Support (METS) developed the Real-time ARV Stock Status (RASS) monitoring tool in 2018. The RASS is a computer-based system integrated with the national health information system that enables weekly tracking of HIV commodity stock levels. The tool provides a dashboard that facilitates the submission of weekly stock status reports, allowing for timely redistribution of commodities and minimising the risk of stock-outs.

In Kampala, HIV-accredited health facilities are required to report ARV and HIV test kit stock status weekly through the dashboard. However, in quarter one 2024, only 4 (12%) of 31 HIV-accredited sites submitted all weekly reports, affecting the timely redistribution of ARVs and test kits. To address this gap, by using a continuous quality improvement approach, we aimed to improve weekly RASS reporting from 0% to 100% by August 2024 at a non-reporting regional referral hospital in Kampala. The selected site was one of the four non-reporting facilities at the time of assessment.

## Research methods and design

### Project design

A continuous quality improvement (CQI) approach was used to enhance weekly HIV commodity reporting using the RASS tool. A CQI approach was used to enhance weekly HIV commodity reporting using the RASS tool. Continuous quality improvement was operationalised through the *Plan–Do–Check–Act (PDCA)* cycle, which provided a structured framework for iterative problem-solving. In the planning phase, the CQI team identified gaps in reporting and designed interventions. The doing phase involved implementing staff orientation and WhatsApp reminders. The checking phase entailed weekly monitoring of reporting compliance using RASS dashboard data, while the acting phase focused on standardising and reinforcing successful interventions to sustain improvements.

To identify barriers to reporting, the team applied the 5 Whys technique: a systematic inquiry method where the question ‘why’ is asked repeatedly until the underlying cause is revealed. For example, when asked why reports were not submitted, the initial answer was that staff were not submitting. Probing further revealed that staff were unfamiliar with RASS procedures, which in turn was because of a lack of orientation for newly transferred staff. This iterative questioning traced the problem to its most modifiable root cause: inadequate orientation.

The 5 Whys analysis was complemented by a fishbone diagram, which categorised contributing factors under personnel, processes, environment, and tools. Personnel-related gaps, particularly a lack of orientation and absence of reminders, emerged as the dominant causes of non-reporting. The CQI team comprised two RASS users, a logistics focal person, a logistics coordinator, and a laboratory coordinator. The logistics focal person led staff orientation and coordinated WhatsApp reminders, while HIV clinic staff supported implementation and monitoring. This ensured accountability and integration of interventions into routine facility operations.

### Project setting

The project was conducted at a regional referral hospital in Kampala, Uganda, an HIV-accredited facility serving approximately 1354 HIV patients as of March 2024. The hospital provides free comprehensive HIV care, including prevention of vertical transmission, antiretroviral therapy (ART), and sexual and gender-based violence services. Despite its critical role in delivering HIV care and treatment services, the hospital faced challenges with accurately and consistently reporting weekly HIV commodities using the RASS system. Despite two logistics officers at this site being trained on RASS reporting during the 2018 national rollout, these challenges persisted.

### Population and sampling strategy

The project involved a CQI team from the hospital’s HIV clinic, including two RASS users, a logistics focal person, a logistics coordinator, and a laboratory coordinator, all previously trained in CQI methods. No sampling was required, as the intervention targeted the entire reporting process at the facility.

### Targeted interventions to enhance real-time antiretroviral stock status reporting

Two interventions were implemented with specific timelines. In April 2024, orientation of two newly transferred RASS users on reporting protocols, including coding, submission deadlines, and verification procedures, was conducted. This orientation was organised and delivered by the hospital’s CQI team, led by the logistics focal person and supported by HIV clinic staff, ensuring that responsibility was clearly assigned and accountability maintained. In May 2024, a WhatsApp group was created to provide weekly reminders. These reminders were coordinated by the logistics focal person, who ensured timely prompts were sent to all RASS users. By embedding these activities within the CQI team’s routine responsibilities, the interventions were not only effective but also positioned for integration into ongoing hospital programmes and Ministry of Health communication channels, thereby enhancing sustainability and scalability.

### Data collection

Baseline data were abstracted from the RASS dashboard in March 2024 to confirm zero weekly reports. Progress was monitored weekly via dashboard data on report submissions. Data extraction was conducted by the hospital’s logistics focal person, who was a trained member of the CQI team. The RASS dashboard itself served as the primary tool, providing standardised weekly reporting templates and automated submission logs. To ensure consistency, the CQI team used predefined indicators (number of reports submitted vs. expected reports per month) and cross-checked entries against the Ministry of Health reporting guidelines. Quality assurance was achieved through double verification: the logistics focal person downloaded and reviewed dashboard outputs, while the CQI coordinator independently validated completeness and accuracy. Any discrepancies were resolved through team review meetings. These procedures ensured that the data collection process was standardised, reliable, and replicable in other HIV-accredited facilities.

### Data analysis

Data were analysed monthly to calculate the percentage of reports submitted, using the formula: numerator = number of reports submitted; denominator = number of expected reports (typically four per month). Descriptive statistics were applied to generate frequencies and percentages of reporting compliance, which were then plotted as monthly trends to visualise progress.

To ensure consistency, the CQI team relied on predefined indicators aligned with the Ministry of Health reporting standards, including timeliness (submission by deadline), completeness (all required data elements entered), and accuracy (cross-checked against facility stock records). Data outputs from the RASS dashboard were exported into Microsoft Excel, where compliance rates were computed and visualised using line graphs.

Quality assurance was achieved through a two-step validation process: the logistics focal person performed the initial calculations, and the CQI coordinator independently reviewed the outputs for accuracy. Any discrepancies were discussed and resolved during weekly CQI meetings. This dual verification process ensured the reliability of the analysis and enhanced confidence in the observed trends.

### Ethical considerations

This project was determined as non-research activity by the Institutional Review Board at a public health institute; approval number: CGH-EAFRB-7/8/24-de2c0. As the project utilised routine programmatic data without personal identifiers, written or oral consent was not required. To ensure data confidentiality, real-time ARV Stock Status (RASS) dashboard data were stored on secure servers with access restricted to authorised personnel only.

## Results

### Baseline assessment at the selected HIV-accredited regional referral hospital in Kampala, March 2024

In March 2024, the selected HIV-accredited regional referral hospital in Kampala submitted zero (0%) of the expected four weekly HIV commodity reports via the RASS system, as confirmed by data abstracted from the RASS dashboard.

### Root cause analysis for non-reporting at the HIV-accredited regional referral hospital, Kampala March 2024

**FIGURE 1:**
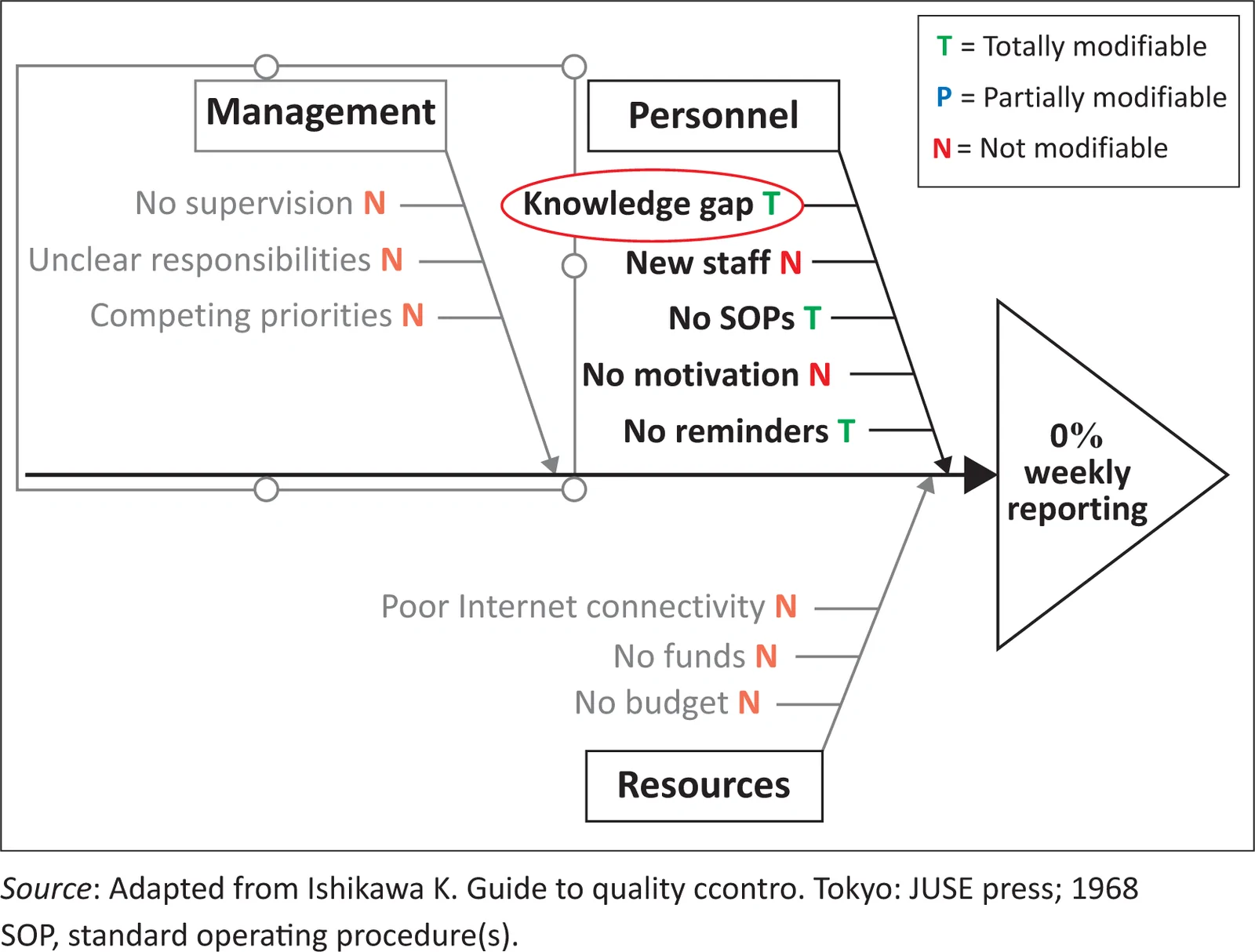
Fishbone diagram illustrating root causes of non-reporting at the selected HIV-accredited regional referral hospital, Kampala, March 2024.

Using the fishbone technique in March 2024, the CQI team identified personnel-related factors as the primary cause of non-reporting. Specifically, the lack of orientation for two newly transferred RASS users was the most modifiable root cause (Figure 1)^4^. The absence of reminders was a secondary contributing factor.

### Effect of targeted interventions on addressing non-reporting of HIV commodities at an HIV-accredited regional referral hospital, March 2024 – August 2024

**FIGURE 2:**
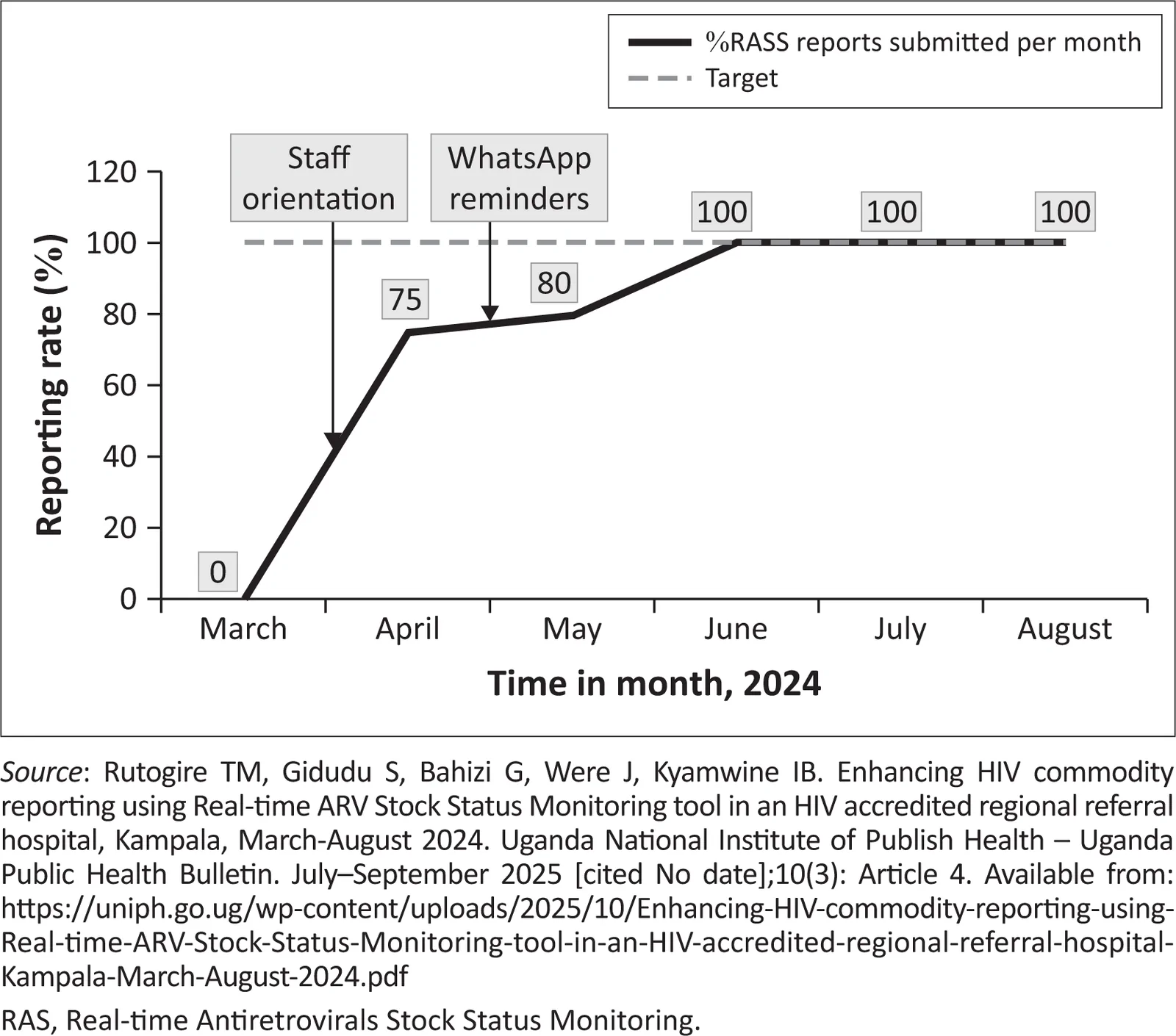
Weekly human immunodeficiency virus commodity reporting rates at a selected HIV-accredited regional referral hospital, Kampala; March 2024 – August 2024.

The interventions led to progressive improvements in weekly reporting rates (Figure 2)^5^. At baseline in March 2024, reporting was 0% (0/4 expected reports submitted). In April 2024, after staff orientation, reporting rose to 75% (*n* = 3/4) reports submitted, with one missed because of a public holiday. In May 2024, introducing WhatsApp reminders further increased reporting to 80% (*n* = 8/10 reports submitted for April and May combined). From June 2024 to August 2024, monitoring confirmed sustained 100% reporting (Figure 2).

## Discussion

This CQI project demonstrated that targeted, low-cost interventions (staff orientation and WhatsApp reminders) significantly improved HIV commodity reporting at the selected HIV-accredited regional referral hospital. The project began with a 2-day staff orientation on 01–02 April 2024, highlighting the critical role of structured training in addressing knowledge gaps, particularly among newly transferred logistics officers. Reporting compliance initially increased from 0% to 75% by the end of April 2024. This finding aligns with existing literature demonstrating that comprehensive employee training enhances performance and operational efficiency in health systems.^6^ The orientation likely addressed deficiencies in RASS system familiarity, enabling users to navigate its technical requirements more effectively, a challenge that had persisted at this site despite training during the 2018 national rollout.

The subsequent introduction of WhatsApp reminders on 03 May 2024 further improved compliance, sustaining 80% reporting by the end of May 2024 and achieving 100% by early June 2024. This success reflects the power of low-cost digital communication tools to reinforce accountability and foster consistent reporting behaviour. WhatsApp was widely used because of its accessibility and affordability. It provided timely prompts that aligned with staff workflows, reducing delay.^7^ This outcome is consistent with studies on text message reminders in healthcare, which demonstrate their efficacy in improving protocol adherence, such as medication adherence and data reporting.^8^ Together, these interventions addressed both technical and behavioural barriers, creating a combined effect that led to sustained improvements.

These findings highlight the importance of addressing personnel-related barriers, such as inadequate orientation and a lack of ongoing support, to improve reporting compliance in resource-constrained settings. The hospital’s ability to achieve 100% reporting compliance by June 2024 suggests that combining training with continuous reinforcement mechanisms can overcome longstanding challenges, such as those observed since the 2018 RASS rollout. The scalability of these interventions offers significant potential for replication across the 31 HIV-accredited sites.

Importantly, these interventions can be institutionalised into existing CQI structures and the Ministry of Health communication channels. Staff orientation can be embedded in induction programmes for newly transferred logistics officers, ensuring that training is consistently delivered. WhatsApp reminders can be integrated into routine Ministry of Health (MoH) communication platforms, providing a sustainable, low-cost mechanism for reinforcing compliance.^9^ By embedding these practices into established systems, the interventions move beyond one-off improvements and become scalable strategies for strengthening HIV commodity reporting across facilities.

Improved RASS reporting could enhance the timeliness and accuracy of HIV commodity data, facilitating efficient ARV redistribution and supporting progress towards the UNAIDS 95-95-95 targets (95% of people living with HIV knowing their status, 95% of diagnosed individuals on ART, and 95% of those on ART achieving viral suppression). By ensuring reliable commodity tracking, these interventions could mitigate stock-outs and overstocking, optimising resource allocation in a high-burden HIV care setting

### Limitations

While our interventions were successful, our project had some limitations. Firstly, we focused on a single HIV-accredited site, which limits the generalisability of our findings to all 31 sites in the region. Secondly, our project did not assess the long-term sustainability of these interventions beyond August 2024.

Thirdly, we did not control for potential confounding factors that may have influenced reporting compliance. For example, external influences such as increased regional supervision, parallel Ministry of Health initiatives or heightened staff motivation unrelated to the interventions could have contributed to the observed improvements. Because this was a quality improvement project rather than a controlled study, attributing the entire effect solely to orientation and WhatsApp reminders should be interpreted with caution.

Future projects should therefore consider designs that account for confounding variables, such as including multiple sites, using comparison groups, or applying more rigorous monitoring frameworks. We recommend institutionalising orientation for new staff and weekly reminders at all HIV-accredited sites to improve HIV commodity reporting, while also evaluating the independent and combined effects of such interventions under controlled conditions.

## Conclusion

Staff orientation and WhatsApp reminders were associated with improved weekly HIV commodity reporting at the selected HIV-accredited site. These interventions addressed personnel-related barriers and supported sustained compliance through August 2024. However, because this was a quality improvement project conducted at a single site without a control group, the improvements observed cannot be attributed solely to the interventions. External factors such as regional supervision or parallel Ministry of Health initiatives may also have contributed.

Despite these limitations, the findings suggest that structured orientation for newly transferred staff and low-cost digital reminders hold promise as practical strategies for strengthening HIV commodity reporting. Further evaluation across multiple sites and under controlled conditions is needed to confirm their effectiveness and assess long-term sustainability. Institutionalising such approaches within routine CQI activities and the Ministry of Health communication channels may offer potential benefits for enhancing HIV commodity stock management and supporting progress towards the UNAIDS 95-95-95 targets.
